# Comparison of Two Different Methods of Fine Needle Aspiration Biopsy and Histopathology for Thyroid Nodules

**DOI:** 10.7759/cureus.6740

**Published:** 2020-01-22

**Authors:** Hakki M Karakas, Gulsah Bicer, Ozge Findik, Ahmet Nedim Kahraman

**Affiliations:** 1 Radiology, University of Health Sciences, Istanbul Fatih Sultan Mehmet Training and Research Hospital, Istanbul, TUR

**Keywords:** thyroid nodules, usg-guided biopsy, cytopathology

## Abstract

Objective: Two different methods for fine needle aspiration biopsy (FNAB) of thyroid nodules (multi-pass conventional smear, MPCS; single-pass liquid-based cytology, SPLBC) were evaluated regarding the magnitude of nondiagnostic/unsatisfactory sampling ratio, and basic demographic and ultrasonographic (USG) factors to predict such outcome.

Methods: One thousand FNAB patients were retrospectively assessed. Of them, 517 nodules were evaluated with the conventional smear method, and the rest were evaluated with liquid-based cytology method using the Bethesda System for Reporting Thyroid Cytopathology. FNAB technique had certain procedural differences for both pathological methods. For conventional smear, a modified "needle-only" technique with three independent passes was performed, whereas a single pass was used for liquid-based cytology. The reduction of nondiagnostic/unsatisfactory results constituted the basis of this study. Pathological results, therefore, were subgrouped under "nondiagnostic/unsatisfactory" (Category I), "benign" (Category II), and "atypia/neoplasia/malignancy" (Category III-VI).

Results: Both FNAB groups were not statistically different or only slightly different regarding size (*P* = 0.196), echogenicity (*P* = 0.014), and the presence of echogenic foci (*P* = 0.11), therefore considered to have equal USG properties. In MPCS method, the nondiagnostic/unsatisfactory rate (i.e., Category I) was 24%. Other cytological results were as follows: Category II (67.1%), Category III-VI (8.8%). In SPLBC method, the nondiagnostic/unsatisfactory rate (i.e., Category I) was 14.5%. Other cytological results were as follows: Category II (77.6%), Category III-VI (7.8%). A significant difference was found between two sampling methods regarding pathological results (Independent samples t-test, *P* < 0.0001). The demographic and USG factors, considered in this study, did not offer a successful prediction of nondiagnostic/unsatisfactory outcomes.

Conclusion: SPLBC has significantly lower (14.5% vs 24%) nondiagnostic rate than MPCS, and higher 77.6% vs 67.1%) Category II rate than MPCS. This may point to the possibility that MPCS method undercategorizes many benign (i.e., Category II) nodules under nondiagnostic/unsatisfactory category. The success of the former is due to the elimination of confounding material during the process. Single pass, also, increases patient comfort and compliance, and has additional advantages for the interventionalist, as it obviates the need to smear aspirates. This dramatically decreases the actual duration of the biopsy procedure and is free of interventionalist expertise for smearing.

## Introduction

Thyroid nodules are not uncommon. Epidemiologic studies have shown the prevalence of palpable thyroid nodules to be approximately 5% in women and 1% in men, living in iodine-sufficient parts of the world [1-2]. Ultrasound (USG) studies can detect thyroid nodules in up to 19%-68% of randomly selected individuals, with higher frequencies in women and the elderly [3-4]. The clinical importance of thyroid nodules rests with the need to exclude thyroid cancer, which occurs in 7%-15% of cases depending on age, sex, previous exposure to ionizing radiation, family history, and other factors [5-6]. The diagnostic work-up of thyroid nodules, therefore, is a definite necessity but is also an economic burden on health systems. 

Thyroid nodules are initially assessed with USG. Using the constellation of USG features, a high-risk nodule may be seen on grayscale USG as a solid and markedly hypoechoic lesion with microcalcifications, a microlobulated or an irregular border, and a shape taller-than-wide. However, none of these features, individually or in combination, are definitive in diagnosis, and it is, therefore, critical to recognize that USG does not replace fine-needle aspiration biopsy (FNAB) evaluation [7]. 

FNAB under USG guidance is a valuable diagnostic tool for characterizing thyroid nodules. There is a worldwide consensus on this method for its simplicity and safety and is regarded as the most accurate and cost-effective method for the selection of surgical patients [8]. However, even this method has a finite diagnostic efficacy. It has a 2.7% inadequacy rate in most experienced hands and after five consecutive passes [9].

Tissue samples taken with FNAB can be evaluated either with a conventional smear or liquid-based cytology or in the combination of both methods. Smear is the conventional preparation method that can be employed in a simple setting. However, in that method, samples, often contaminated by blood and/or USG transmission gel, maybe "inadequate." To overcome that limitation, multiple passes, almost up to five, are usually performed. However, multiple passes increase procedural time and patient discomfort. Liquid-based cytology is a relatively novel method that is based on the use of a semi-automated device to process cellular aspirates. The method theoretically requires lower passes and does not get confounded by blood and/or transmission gel. Therefore, FNAB procedure to be employed is largely dependent on the subsequent pathological evaluation method and shows clear differences regarding the number of passes, the speed of sampling, the negative pressure to be applied to the syringe, etc.

The purpose of the study was to compare the diagnostic success of the above-mentioned methods of FNAB and pathological evaluation for thyroid nodules (i.e., multi-pass conventional smear method (MPCS) vs. single-pass liquid-based cytology method, SPLBC) regarding nondiagnostic/insufficient sampling. Basic demographic (patient age and sex) and USG (nodule's size, echogenicity, composition, and the presence of echogenic foci) factors that may affect the technical success rate were also evaluated to predict inadequate sampling. 

## Materials and methods

The study was conducted in the non-vascular interventional radiology unit. Interventional procedures pertaining to this study were performed in a nine-month period in the same setting and by the same experienced interventionalist. 

Patients

Because of the institutional decision, department of pathology has changed its practice from conventional smears to liquid-based cytology. For the purpose of the study, 517 patients that were biopsied before that practice change, and 483 patients that were biopsied after that have constituted the study sample. 

As stated above, different patients were used to investigate two different FNAB techniques, instead of sampling the same nodule with both methods. The reason behind that experimental design was mainly due to ethical consideration, patient compliance, and national reimbursement policy.

The study group consisted of 175 males with ages between 20 and 88 (56 ± 13) and 825 females with ages between 14 and 91 (51 ± 13). USG-guided FNABs have been requested by departments of endocrinology and/or general surgery according to patients’ previous clinical and radiological findings.

For each patient, FNAB of only one nodule was taken into the study and that nodule was the first to be sampled for that patient. This retrospective study, therefore, included 1000 thyroid nodules. 

USG evaluation

Each nodule was radiologically investigated with USG (Aplio^TM^ 500 Platinum, Toshiba Medical, Japan) using a 7.5 MHz broadband linear array transducer at the beginning of biopsy procedure. Nodule's composition, echogenicity, size, and the presence of echogenic foci were key features to be evaluated. Nodule composition was assessed under three categories as (1) cystic or almost cystic or spongiform, (2) mixed cystic and solid, and (3) solid or almost completely solid. Nodule's echogenicity was assessed under three categories (1) hypoechoic, (2) isoechoic, and (3) hyperechoic. Nodule's size was determined as the mean of two greatest diameters perpendicular to each other. The presence of echogenic foci was assessed under four categories as (1) none or large comet tail artifact, (2) presence of macrocalcifications, (3) presence of peripheral calcifications, and (4) presence of punctate calcifications.

FNAB technique

The procedure was guided under the same USG system. FNAB was performed using a 10 mL syringe and 21 G needle. The syringe was placed in Cameco syringe pistol and negative pressure was alleviated before skin entry. The perpendicular technique was employed where nodules were positioned in the mid-portion of the screen and the point of needle insertion was central, just over the nodule to be targeted. The needle was advanced perpendicular to the transducer’s footprint. The needle tip was continuously monitored on the USG screen during the entire procedure to avoid any complication and to avoid cystic components to assure adequate sampling. In cases where cystic or almost cystic nodules were present, cystic components were carefully evacuated, and sampling was subsequently performed from the remaining solid component. Local anesthesia was not applied in any case. 

For the MPCS method, a modified "needle-only" (i.e., Zajdela) technique was used. In this technique, no aspiration or negative pressure was utilized, and very rapid down and up motion was performed to prevent the movement of blood into the needle [10]. Cells were detached by the cutting edge of the needle and were conducted into the lumen mainly by capillary force. Using this technique, at least two consecutive passes were performed for each nodule. In case of the presence of blood-stained material, a third and the last pass was performed. The extracted material was expelled onto a glass slide, spread, and then immediately fixed by immersing it in absolute ethanol for fixation.

For the SPLBC method, a single pass was performed for each nodule. In that method, the needle tip was advanced and withdrawn several times under negative pressure to assure adequate sampling of the nodule. The extracted material was then injected into falcon tubes which contain 7 mL methanol-based fixative (ThinPrep®^)^ CytoLyt® Solution, Hologic®​​​​, USA).

Cytological evaluation

**Figure 1:**
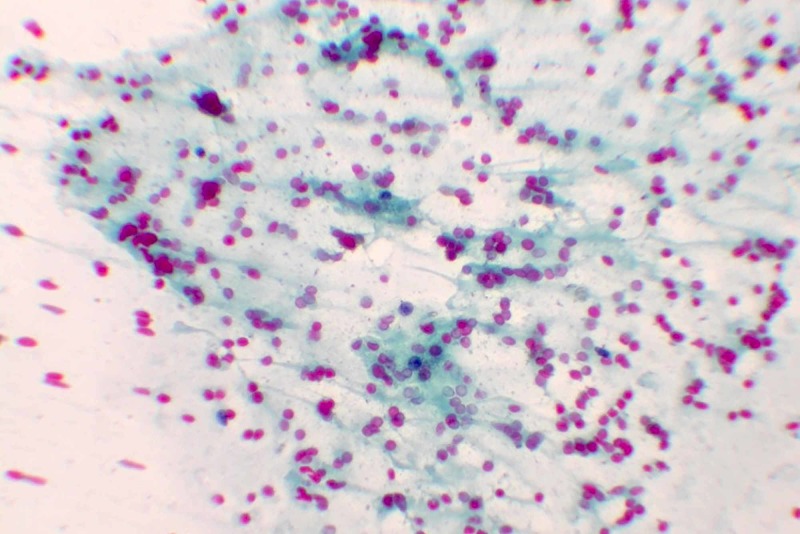
Nondiagnostic conventional smear. The background is occupied with erythrocytes and proteinous material that complicate pathological evaluation. The sample belongs to a patient from the study.

**Figure 2:**
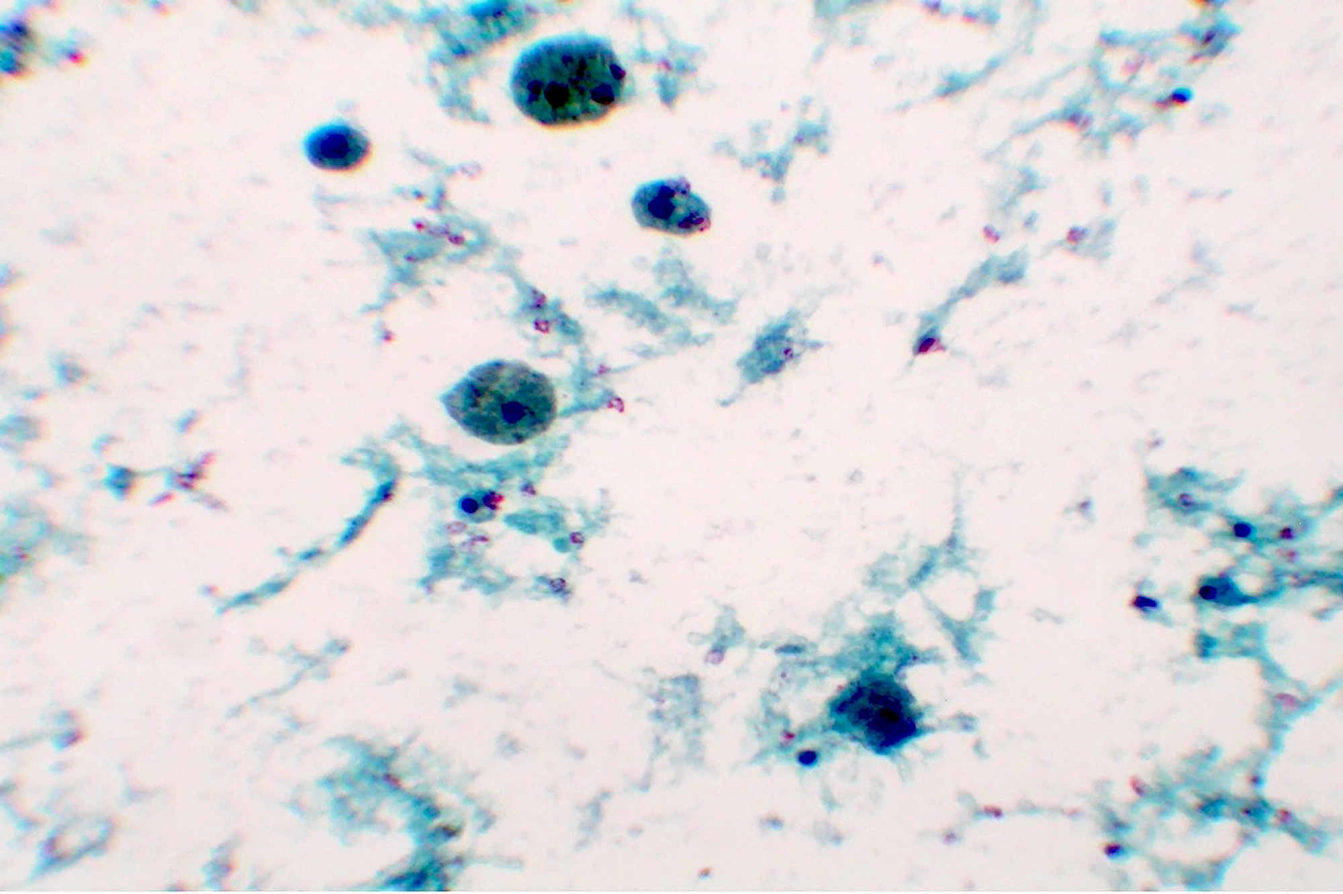
Nondiagnostic liquid-based cytology. The background is clear but there is no evidence of benign or neoplastic cells except hemosiderin-laden macrophages. The sample belongs to another patient from the study.

All materials were assessed by pathologists who had over 10 years of experience. The Bethesda System for Reporting Thyroid Cytopathology (TBSRTC) was used for pathological classification [11]. According to that system, every thyroid FNA report should begin with one of six diagnostic categories: (I) nondiagnostic or unsatisfactory; (II) benign; (III) atypia of undetermined significance (AUS) or follicular lesion of undetermined significance (FLUS); (IV) follicular neoplasm or suspicious for a follicular neoplasm; (V) suspicious for malignancy; and (VI) malignant. As stated above, Category I represents mainly nondiagnostic or unsatisfactory specimens; mainly due to cyst fluid only, virtually acellular specimen, or other confounding factors such as obscuring blood, clotting artifact, etc. (Figure 1 and Figure 2). The reduction of the rate of this category of nondiagnostic or unsatisfactory results was constituted the basis of this study. The study group, therefore, was divided into three subgroups: "Nondiagnostic/unsatisfactory" (i.e., Category I), "Benign" (i.e., Category II), and "Atypia/neoplasia/malignancy" (i.e., Category III-VI).

Statistical analyses

Statistical evaluation was performed using IBM® SPSS® Statistics (Version 25, IBM®, USA), and G*Power (Release 3.1.9.5, Heinrich Heine Universitat Dusseldorf, Germany). Data were described using descriptive statistical methods. Independent samples t-test was used as a parametric, and chi-square test was used as a nonparametric test to explore differences between study groups regarding USG features. Independent samples t-test was also employed to explore the significance of the difference between two sampling methods regarding ordinal pathological results. Logistic regression was employed on both groups to predict the rate of unsatisfactory samples before the actual pathological evaluation. In that analysis, possible influential categorical (patient's sex, nodule's composition, echogenicity, presence of echogenic foci), and numerical (patient's age, nodule's size) were taken into account. All *P* values were reported in an opened form and *P* < 0.05 was chosen as the level of significance. For the statistical design that was described above, the required sample size was 210 patients. By using the actual sample size of 1000 patients, the statistical power was equal to 1.00.

## Results

The size of the nodules that were evaluated with MPCS method was between 4 and 38 mm (mean: 13.6, SD: 6.3). For SPLBC, the nodules were between 3 and 47 mm (mean: 14.1, SD: 6.9) in size. Equality of variances was tested with Levene's test (*F* = 0.913, *P* = 0.340). Both groups were not found to be significantly different regarding this variable (independent samples t-test, *P* = 0.196) (Table 1).

For MPCS method, nodules were found to be hypoechoic in 238 (46%), isoechoic in 247 (47.8%), and hyperechoic in 32 (6.2%) of the cases. For SPLBC method, nodules were found to be hypoechoic in 196 (40.4%), isoechoic in 270 (55.9%), and hyperechoic in 17 (3.5%) of the cases. Both groups were significantly different regarding this variable (Pearson Chi-Square, *P* = 0.014) (Table 1).

For MPCS method, nodules were found to be cystic or almost cystic or spongiform in 19 (3.7%), mixed cystic and solid in 224 (43.3%), and solid or almost completely solid in 274 (53%) of the cases. For SPLBC method, nodules were found to be cystic or almost cystic or spongiform in 5 (1%), mixed cystic and solid in 156 (32.3%), and solid or almost completely solid in 322 (66.7%) of the cases. Both groups were significantly different regarding this variable (Pearson chi-square, *P* < 0.0001). However, the confounding effect of cystic components on biopsy results was eliminated as described above (Table 1).

For MPCS method, nodules were found to have no calcification or have comet tail artifact in 449 (86.8%), macrocalcifications in 18 (3.5%), peripheral calcifications in 8 (1.5%), and punctate calcifications in 42 (8.1%) of the cases. For SPLBC method, nodules were found to have no calcification or have comet tail artifact in 399 (82.6%), macrocalcifications in 30 (6.2%), peripheral calcifications in 5 (1%), and punctate calcifications in 49 (10.1%) of the cases. Both groups were not significantly different regarding this variable (Pearson chi-square, *P* = 0.11) (Table 1).

Out of 1000 patients that were analyzed for this study, only one had experienced procedure-related hemorrhage. The hemorrhage was measured as 15 mm in thickness and was self-limiting. None of the patients had reported excessive pain that required analgesic administration. Therefore, groups were not statistically compared regarding complication rates.

**Figure 3:**
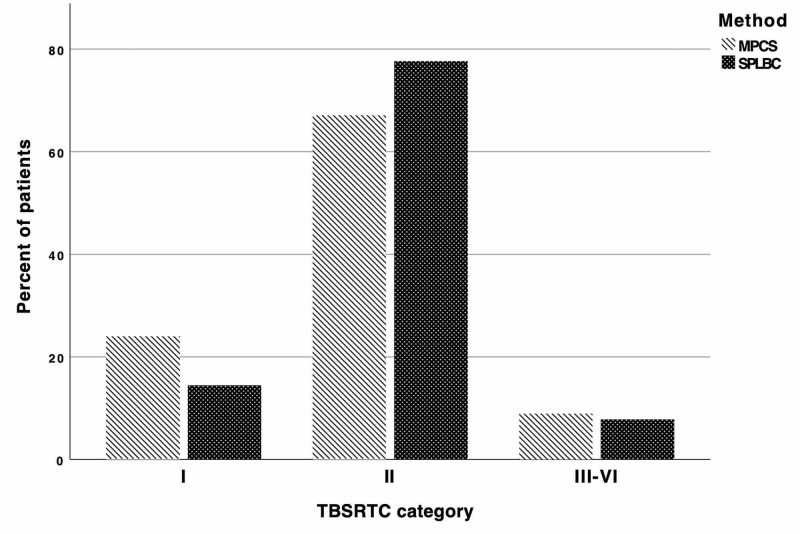
Percent distribution of patients according to their The Bethesda System for Reporting Thyroid Cytopathology categories for both method of fine needle aspiration biopsy and cytopathology.

In MPCS method, the nondiagnostic/unsatisfactory rate (i.e., Category I) was 24%. Other cytological results were as follows: Category II (67.1%), Category III-VI (8.8%). In SPLBC method, the nondiagnostic/unsatisfactory rate (i.e., Category I) was 14.5%. Other cytological results were as follows: Category II (77.6%), Category III-VI (7.8%) (Figure 3). There was a statistically significant difference between the two sampling methods regarding pathological results (Independent samples t-test, *P* < 0.0001) (Table 1).

 

**Table 1:** Descriptive statistics of ultrasound and histopathological findings and statistical significance of their differences between different sampling methods Data were presented as n (%) or mean (min-max).  US ultrasound, TBSRTC; The Bethesda System for Reporting Thyroid Cytopathology; MPCS, multi-pass conventional smear, SPLBC, single-pass liquid-based cytology.

Variable	Sampling method		Total	P
	MPCS	SPLBC		
Size in US				
Diameter	13.6 (4-38)	14.1 (3-47)	13.8 (3-47)	0.196
Echogenecity in US				
Hypoechoic	238 (46))	196 (40.4)	434 (43.4)	0.014
Isoechoic	247 (47.8)	270 (55.9)	517 (51.7)	
Hyperechoic	32 (6.2)	17 (3.5)	49 (4.9)	
Composition in US				
Cystic/almost cystic/spongiform	19 (3.7)	5 (1)	24 (2.4)	<0.0001
Mixed cystic and solid	224 (43.3)	156 (32.3)	380 (38)	
Solid/almost completely solid	274 (53)	322 (66.7)	596 (59.6)	
Echogenic foci in US				
No calcification or comet tail	449 (86.8)	399 (82.6)	848 (84.8)	0.11
Macrocalcifications	18 (3.5)	30 (6.2)	48 (4.8)	
Peripheral calcifications	8 (1.5)	5 (1)	13 (1.3)	
Punctate calcifications	42 (8.1)	49 (10.1)	91 (9.1)	
Category in TBSRTC				
I	124 (24)	70 (14.5)	194 (19.4)	<0.0001
II	347 (67.1)	375 (77.6)	722 (72.2)	
III-VI	46 (8.9)	38 (7.9)	84 (8.4)	

The patient's age and sex, and nodule's echogenicity, mean size, composition, echogenicity, and the presence of echogenic foci were evaluated as predictive factors for nondiagnostic sampling. For MPCS, only age (*P* = 0.018), sex (*P* = 0.001), and nodule's size (*P* < 0.000) were significantly different. However, the combination of these factors offered a successful prediction of Category I (nondiagnostic or unsatisfactory) results in only 7.3% of the cases for this method. For SPLBC, only echogenecity was significantly different (*P* = 0.001). However, this factor did not provide a successful prediction of Category I (nondiagnostic or unsatisfactory) results in any of the cases for this method. 

## Discussion

In TBSRTC, inadequate samples are pathologically reported as Category I. This category applies to samples that are nondiagnostic or unsatisfactory due to obscuring blood, overly thick smears, air drying of alcohol-fixed smears, or an inadequate number of follicular cells. Nondiagnostic or unsatisfactory results cause great anxiety to the operator (i.e., performing interventional radiologist) and/or the patient. This anxiety mainly stems from the fact that the risk of malignancy for a single nondiagnostic aspirate is around 20% [12]. The risk of malignancy may be reduced by repeating the procedure [12]. However, repeated interventions add extra costs to the care cycle, and disturb patients psychologically.

In this study, nondiagnostic or unsatisfactory biopsy rate was 24% for the MPCS method and 14.5% for the SPLBC method. The nondiagnostic rate for USG-guided FNABs varies widely among different studies, with rates as high as 47% and as low as 0.6%. The latter value belongs to studies with on-site cytology [13-14]. However, in the majority of the studies, the nondiagnostic rate usually ranges between 8% and 20% [15-16], which are compatible with the average nondiagnostic FNAB rate (19.25%) of our study.

We were unable to predict Category I lesions, a priori, using basic demographic data of the patients and/or basic grayscale USG features. Although there are other factors that were shown in previous studies to influence nondiagnostic rates for FNAB results, including the skill of the operator, vascularity of the nodule, and the cystic component of the nodule [17-18], the most important factor influencing the negative results stems from the presence of cells admixed with debris, blood, and exudates, which make the interpretation difficult. The latter factor leads to a high proportion of cases reported as inadequate or unsatisfactory for assessment [19]. Although, the Zajdela technique, the most preferred maneuver in MPCS method, was originally developed to reduce aspiration trauma to cells, and to increase the quality of the cytological smears by providing less blood in the samples, the handling of the needle needs a certain expertise even for experienced radiologists, as it is practiced with a sensitive and a very rapid wrist movement [20]. Liquid-based cytology method, on the other hand, does not necessitate such a manipulation, and is superior to conventional smear method, as it provides a clear background, monolayer cell preparation, and excellent cell preservation. Therefore, it is easier and less time consuming to obtain and to screen and interpret such materials. 

As mentioned above, SPLBC method had a lower nondiagnostic sampling rate than the MPCS method (i.e., 14.5% vs. 24%). The absolute difference between both methods for Category I nodules (9.5%) and the difference between both methods for Category II nodules (10.5%) was almost equal. This equality may point to the possibility that the MPCS method undercategorizes many benign (i.e., category II) nodules as nondiagnostic/unsatisfactory. The difference between both methods for Category III-VI nodules (1%), on the other hand, was not significant. According to the above-mentioned observations, benign lesions possibly constitute the bulk of nondiagnostic studies in MPCS method. 

The main limitation of the study was the use of two independent groups. As such, different patients were used to investigate two different FNAB techniques, instead of sampling the same nodule with both methods. The reason behind that experimental design was mainly due to ethical consideration, patient compliance, and national reimbursement policy. However, the existence of a large number of samples, and the exclusion of the operator factor, partially deemphasizes that limitation.

SPLBC method is more expensive than the conventional smear method [19]. On that context, its total cost is almost twice as much as the cost of MPCS method in our institution. However, this economical load may be counterbalanced by the fact that the former method only requires a single pass, hence, decreases the actual duration of the biopsy procedure by two-fold, increases patient comfort and compliance, and decreases complications. It also provides high-quality samples that decrease false-negative results. Nondiagnostic/Unsatisfactory result rate in MPCS method may potentially be lowered by rapid on-site evaluation (ROSE), a cytological assessment performed on-site during FNAB procedure to assess the sample acceptability [21]. However, ROSE requires the on-site availability and the allocation of cytopathologists and substantially increases the overall costs. The MPCS method, on the other hand, causes less pain than the SPLBC method due to the aspiration step, but it requires certain expertise.

## Conclusions

The lower re-biopsy rates of the SPLBC method have tangible economic benefits when the very high incidence of thyroid nodules is considered. The method has additional advantages for the interventionalist as it obviates the need to smear aspirates. This dramatically decreases the actual duration of the biopsy procedure and is free of interventionalist expertise for smearing. SPLBC method, therefore, should be preferred when sampling thyroid nodules.
